# Maternal C-Peptide and Insulin Sensitivity, but Not BMI, Associate with Fatty Acids in the First Trimester of Pregnancy

**DOI:** 10.3390/ijms221910422

**Published:** 2021-09-27

**Authors:** Julia Bandres-Meriz, Alejandro Majali-Martinez, Denise Hoch, Milagros Morante, Andreas Glasner, Mireille N. M. van Poppel, Gernot Desoye, Emilio Herrera

**Affiliations:** 1 Department of Obstetrics and Gynaecology, Medical University of Graz, 8036 Graz, Austria; a.majali-martinez@medunigraz.at (A.M.-M.); denise.hoch@medunigraz.at (D.H.); 2 Faculty of Pharmacy, Universidad San Pablo CEU, 28668 Madrid, Spain; morantesantana@gmail.com (M.M.); eherrera@ceu.es (E.H.); 3 Femina Med-Center, 8010 Graz, Austria; office@dr-glasner.at; 4 Institute of Human Movement Science, Sport and Health, University of Graz, 8010 Graz, Austria; mireille.van-poppel@uni-graz.at

**Keywords:** early pregnancy, obesity, fatty acids, glucose, C-peptide, insulin sensitivity, fetal sex

## Abstract

Maternal obesity in pregnancy is a pro-inflammatory condition exposing the fetus to an adverse environment. Here, we tested associations of maternal obesity (primary exposures: BMI, leptin) and metabolic parameters (secondary exposures: glucose, C-peptide, and insulin sensitivity) with total serum concentrations of fatty acids in the first trimester of human pregnancy. This cross-sectional study included 123 non-smoking women with singleton pregnancy. In maternal serum, cotinine, leptin, and C-peptide (ELISA), glucose (hexokinase-based test) and fatty acids (gas chromatography) were quantified, and the insulin sensitivity index (IS_HOMA_) was calculated. Concentrations of fatty acid classes and total fatty acids did not differ between BMI or leptin categories. However, *n*-3 polyunsaturated fatty acids (PUFA) were decreased in the category with the highest C-peptide concentration (*n*-3 PUFA: CI −35.82–−6.28, *p* < 0.006) and in the lowest IS_HOMA_ category (*n*-3 PUFA: CI −36.48–−5.61, *p* < 0.008). In a subcohort, in which fetal sex was determined (RT-qPCR of placental tissue), C-peptide was significantly associated with docosahexaenoic acid (DHA) in mothers bearing a female (n = 46), but not male (n = 37) fetus. In conclusion, pregnant women with high fasting C-peptide and low IS_HOMA_ had decreased *n*-3 PUFA, and DHA was lower with higher C-peptide only in mothers bearing a female fetus.

## 1. Introduction

The prevalence of obesity in women of reproductive age is increasing worldwide. In pregnancy, obesity exposes the fetus to an adverse intrauterine environment. This often results in short- and long-term health consequences, including altered fetal neurodevelopment, increased fetal growth, increased neonatal adiposity, and a higher risk for the development of cognitive and behavioral disorders and obesity early in life [1,2,3,4].

Women who are obese frequently develop gestational diabetes mellitus (GDM) in pregnancy [5] and both obesity and diabetes are characterized by chronic low-grade inflammation [6].

While the strong effect of hyperglycemia on fetal growth has been well established [7,8], there are increasing arguments suggesting that triglycerides and non-esterified fatty acids (NEFA) also contribute to fetal overgrowth [9,10]. Indeed, it has been proposed that altered lipid metabolism rather than hyperglycemia constitutes a risk for macrosomia in GDM [11]. Likewise, maternal long-chain polyunsaturated fatty acids (PUFA) have been associated with cognitive and behavioral performance in childhood [12], and decreased maternal *n*-3 PUFA concentrations correlate with lower brain volume in the offspring [13].

Maternal lipid metabolism changes profoundly during pregnancy [14]. Early pregnancy is an anabolic state with increased storage of fat in the mother that will serve as a source of nutrients for the fetus later in pregnancy when the mother is in a catabolic state [15,16]. Maternal lipids, especially fatty acids, are important contributors to building up maternal fat stores and to fetal adiposity. Alterations of fatty acid concentrations in the second half of pregnancy by conditions such as maternal obesity have been well recognized [17,18]. However, to the best of our knowledge, the effects of maternal obesity on fatty acids and the associated changes in the glucose–insulin axis [19] in the first trimester of pregnancy have not been investigated yet. 

In the present study, we tested the potential association of maternal obesity (primary exposures: BMI, leptin) with fasting fatty acids concentrations in the first trimester of human pregnancy. Because obesity is a heterogeneous condition with a proportion of subjects having normal metabolism [20], we also included metabolic parameters (secondary exposures: glucose, C-peptide, and IS_HOMA_) to delineate potential metabolic pathways. Increasing evidence suggests a contribution of fetal sex to maternal metabolism [21,22,23]. To assess potential interactions between fetal sex and maternal metabolic parameters, we performed secondary analyses in a subcohort in which fetal sex was determined.

## 2. Results

### 2.1. Maternal BMI and Leptin Do Not Associate with Fatty Acid Concentrations

**Figure 1:**
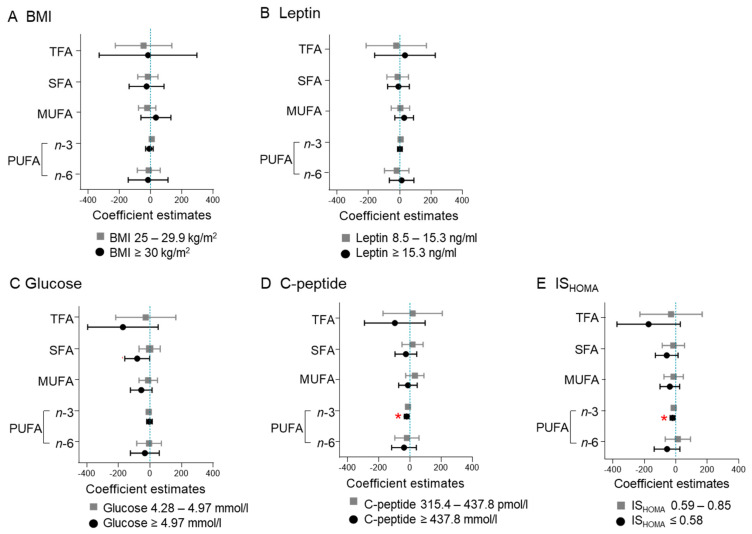
Maternal C-peptide and IS_HOMA_, but not maternal BMI or leptin, associate with *n*-3 PUFA. Measures of maternal obesity (BMI) (**A**), adiposity (leptin) (**B**) and of the glucose–insulin axis (glucose, C-peptide, and IS_HOMA_) (**C**–**E**) were categorized into tertiles and the estimates compared to the reference category: BMI < 25 kg/m^2^, leptin < 8.5 ng/mL, glucose < 4.28 mmol/L, C-peptide < 315.4 pmol/L, IS_HOMA_ > 0.85. SFA: sum of 14:0, 16:0, and 18:0; MUFA: sum of 16:1, 18:1, and 22:1; *n*-3 PUFA: sum of 18:3, 20:3, 20:5, 22:5, and 22:6; *n*-6 PUFA: sum of 18:2, 18:3, 20:3, 20:4, 22:2, 22:4, and 22:5. Model adjusted for gestational age (days), maternal age (years) and processing time (minutes). * Statistically significant difference after correction for multiple testing (FDR = 0.2).

The concentration of total fatty acids, SFA, MUFA, *n*-3 PUFA, or *n*-6 PUFA did not differ between BMI or leptin groups (Figure 1A). Among the individual fatty acids, myristic acid (C 14:0) was decreased (β: −0.20, CI: −0.39–−0.01, *p* = 0.037) in the overweight group (BMI 25–29.9 kg/m^2^) compared to the referent category (BMI < 25 kg/m^2^) (Table S1), which was not statistically significant after correcting for multiple testing. No differences were found between leptin categories, with the exception of dihomo-ƴ-linolenic acid (C 20:3 *n*-6), that was increased (β: 6.22, CI: 0.19–12.25, *p* = 0.043) in the group with high leptin (leptin ≥ 15.3 ng/mL) compared to the referent group (leptin < 8.5 ng/mL). Again, the association lost statistical significance after correcting for multiple testing (Figure 1B, Table S1). 

### 2.2. High Glucose Associates with Decreased SFA Concentration

SFAs were decreased (β: −80.54, CI: −159.29–−1.79, *p* = 0.045) in the tertile with the highest glucose concentration (≥4.97 mmol/L) compared to the referent category (<4.28 mmol/L), but the association was not significant after correcting for multiple testing (Figure 1C). Palmitic acid (C 16:0) was the most abundant (67.1%) SFA and it was significantly decreased in the tertile with the highest glucose concentration (β: −0.11, CI: −0.22–−0.01, *p* = 0.032) (Table S1, Figure S1). However, the association lost statistical significance after correcting for multiple testing.

### 2.3. High C-Peptide and Low IS_HOMA_ Associate with Decreased n-3 PUFA Concentration

The tertile with the highest C-peptide (≥437.8 pmol/L) had decreased (β: −21.05, CI: −35.82–−6.28, *p* = 0.006) *n*-3 PUFA concentration compared to the referent category (C-peptide < 315.4 pmol/L) (Figure 1D). The tertile with the lowest insulin sensitivity (IS_HOMA_ ≤ 0.59) also had lower *n*-3 PUFA concentrations (β: −21.05, CI: −36.48–−5.61, *p* = 0.008) compared to the referent category (IS _HOMA_ > 0.85) (Figure 1E). Both associations remained significant after correcting for multiple testing.

**Figure 2:**
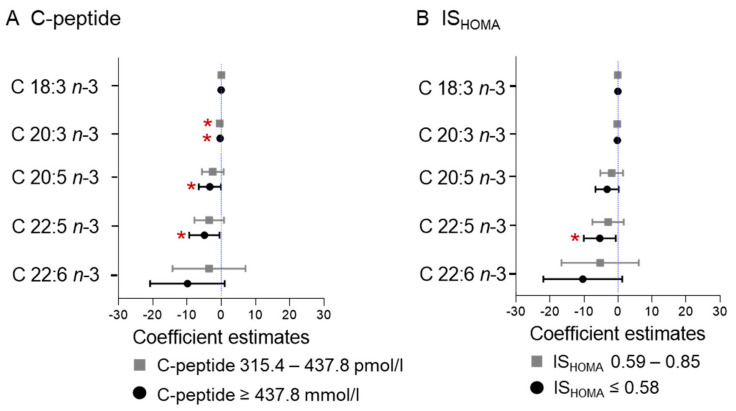
Association of C-peptide and IS_HOMA_ with individual *n*-3 fatty acids. Measures of maternal C-peptide (**A**) and IS_HOMA_ (**B**) were categorized into tertiles and the estimates compared to the reference category: C-peptide < 315.4 pmol/L, ISHOMA > 0.85. Model adjusted for gestational age (days), maternal age (years), and processing time (minutes). * Statistically significant difference after correction for multiple testing (FDR = 0.2).

Next, we wanted to identify the individual fatty acids associating with C-peptide and IS_HOMA_, respectively. Eicosatrienoic acid (C 20:3 *n*-3; 3.1% of total *n*-3 PUFAs, *p* = 0.008), eicosapentaenoic acid (C 20:5 *n*-3; 10.8% of total *n*-3 PUFAs, *p* = 0.043), and docosapentaenoic acid (C 22:5 *n*-3; 16.7% of total *n*-3 PUFAs, *p* = 0.029) concentrations were decreased in the tertile with the highest C-peptide (≥437.8 pmol/L). Based on β estimates, C-peptide had the strongest association with docosapentaenoic acid (C 22:5 *n*-3). Eicosatrienoic acid (C 20:3 *n*-3) was also decreased in the 2nd tertile (C-peptide 315.4–437.8 pmol/L *p* = 0.001) (Figure 2A). Docosapentaenoic acid (C 22:5 *n*-3; *p* = 0.026) was also significantly decreased in the low IS_HOMA_ group (IS_HOMA_ ≤ 0.59) (Figure 2B). All those associations remained significant after adjusting for multiple testing (adjustment including all tests performed between C-peptide and IS_HOMA_ and the individual *n*-3 PUFA; n = 20) (Figure 2).

### 2.4. High C-Peptide and Low IS_HOMA_ Associate with *n*-6/*n*-3 PUFA Ratio

Fasting serum C-peptide (continuous) was significantly associated with the *n*-6/*n*-3 PUFA ratio (β: 0.69, CI: 0.12–1.45, *p* = 0.018) (Table 1). IS_HOMA_ (continuous) was inversely associated with the *n*-6/*n*-3 PUFA ratio (β: −0.59, CI: −1.09–0.87, *p* = 0.022) (Table 1), also after correction for multiple testing.

### 2.5. Indices of Desaturase Activities

None of the metabolic parameters considered in this study (BMI, leptin, glucose, C-peptide and IS_HOMA_) were associated with the ratios palmitoleic acid (C 16:1)/palmitic acid (C 16:0), ƴ-linolenic acid (C 18:3 *n*-6)/linoleic acid (C 18:2 *n*-6), and arachidonic acid (C 20:4)/dihomo-ƴ-linolenic acid (C 20:3) representing the indices of Δ9- Δ6- and Δ5-desaturase activity, respectively. The ratio of arachidonic acid (C 20:4 *n*-6)/linoleic acid (C 18:2 *n*-6) reflecting combined elongase, Δ5-, and Δ6- indices of desaturase activity was positively associated with BMI (continuous) (B: 1.16 CI: 0.09–0.22, *p* = 0.00001) after correcting for multiple testing (not shown). 

### 2.6. Fetal Sex 

We determined fetal sex whenever placental tissue was available (n = 83). Gestational age and maternal metabolic parameters (age, BMI, leptin, glucose, C-peptide, IS_HOMA_, and fatty acids concentration) did not statistically differ between this subgroup (female n = 46, male n = 37) and the total cohort (Table S2). Maternal BMI was significantly (*p* = 0.021) higher in female-bearing mothers compared to male-bearing fetuses (Table S4). 

**Figure 3:**
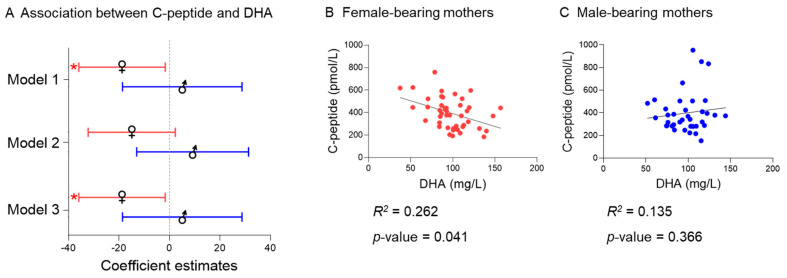
The association between C-peptide and docosahexaenoic acid is specific for female-bearing mothers. (**A**) Representation of the association between C-peptide and docosahexaenoic acid (DHA) in the female (n = 46) and male (n = 37) subgroups (Model 1: Adjusted for gestational age, maternal age and processing time, Model 2: Model 1 + Adjustment for BMI; Model 3: Model 1 + Adjustment for leptin). (**B**) Correlation between C-peptide and docosahexaenoic acid in the female subgroup. (**C**) Correlation between C-peptide and docosahexaenoic acid in the male subgroup. * Statistically significant difference after correction for multiple testing (FDR = 0.2).

There was a significant interaction between fetal sex and BMI (*p* = 0.076), fetal sex and C-peptide (*p* = 0.072), and fetal sex and IS_HOMA_ (*p* < 0.070) in the model for total *n*-3 PUFA, that remained significant after correcting for multiple testing. Therefore, we analyzed associations separately for women bearing a female or a male fetus. However, no significant associations between BMI, C-peptide, or ISHOMA with total *n*-3 PUFA concentration were found in female-bearing mothers nor in the male-bearing mothers. Among the individual *n*-3 PUFA, there was a significant interaction (*p* < 0.1) between fetal sex and C-peptide in the docosahexaenoic acid model (C 22:6 *n*-3), also after adjusting for multiple testing. Further subanalyses showed that the association between C-peptide and docosahexaenoic acid was specific for the female-bearing mothers (Figure 3). Adjusting for BMI or leptin did not significantly change the effect size (model 1: 23.3% model 2: 24.3% model 3: 24.1%) (Table S5). No significant interactions (all *p >* 0.1) were found between fetal sex and the exposure variables (BMI, leptin, glucose, C-peptide, or IS_HOMA_) in the SFA, MUFA, or *n*-6 PUFA models. 

### 2.7. Sensitivity Analysis Excluding Women with High Fasting Glucose

Women with known co-morbidities, e.g., diagnosed with diabetes, were excluded from the study. However, two women included in the study had fasting serum glucose concentration > 7 mmol/L [24] (7.2 mmol/L and 8 mmol/L, respectively). To rule out a potential driving effect of these women on the results, we performed a sensitivity analysis excluding these values and re-analyzing the data. The analyses provided comparable results (Table S2 and Figure S2). The associations between BMI, leptin, C-peptide, and IS_HOMA_ with the fatty acid classes and the individual fatty acids also provided comparable results after excluding these women. 

## 3. Discussion

In the present study, we aimed to test if maternal obesity and adiposity, characterized by BMI and circulating levels of leptin, alter serum fatty acid concentrations already during the first trimester of pregnancy in non-smoking women. We also aimed to identify if potential associations exist between maternal fasting glucose, C-peptide, and IS_HOMA_ and serum fatty acids in this cohort. We found that higher C-peptide and lower IS_HOMA_, but not BMI or leptin, were associated with lower serum total *n*-3 PUFA concentrations in the first trimester of pregnancy. Further sub-analyses considering fetal sex showed an association between C-peptide and docosahexaenoic acid (DHA, C 22:6 *n*-3) specific for the female-bearing mothers. 

Outside of pregnancy, obesity often associates with hyperinsulinemia and we also recently observed an association between obesity and C-peptide in the first trimester of pregnancy [19]. In the present study, BMI was associated with C-peptide, but not with fatty acid concentrations. The association between C-peptide and *n*-3 PUFA concentration was independent of BMI since the adjustments barely changed β-estimates and effect size. Adjusting for maternal leptin as a proxy for fat mass resulted in even stronger associations. 

Obesity is a heterogeneous condition and up to about 30% of the obese adult population are metabolically normal with normal lipids and HOMA-insulin resistance [20]. Our study was not designed to distinguish between metabolically normal and abnormal obese women. Therefore, we cannot exclude that such heterogeneity in our cohort, if present at all, may have masked a potential BMI association with the fatty acids. However, the results suggest that using obesity proxies such as BMI might not be well suited to understand metabolism in the first trimester of pregnancy.

Decreased proportions of *n*-3 PUFA in obese children [25] and adults [26] have been previously reported. In an observational case-control study [25] comprising 67 obese and 67 normal-weight children (8–12 years), plasma *n*-3 PUFA and docosahexaenoic acid (DHA, C 22:6 *n*-3) were lower in the highly obese children despite increased consumption (grams and %) of *n*-3 PUFA in this group. Similar to our study, the concentration of the essential fatty acid α-linolenic acid (C 18:3 *n*-3) was not significantly different between groups, suggesting that a dysregulation in the desaturation and elongation steps might take place in obese individuals. In the same study, obese children had significantly higher insulin concentrations, which raises the question of whether the observed changes in *n*-3 PUFA metabolism may be attributed to the hyperinsulinemia associated with obesity. Indeed, outside pregnancy, *n*-3 PUFA supplementation has been shown to improve insulin sensitivity without changes in BMI [27]. In a rat model, *n*-3 PUFA supplementation inhibited toll-like receptor 4 (TLR4) activation in the muscle and resulted in improved insulin sensitivity [28], suggesting that *n*-3 PUFA exert anti-inflammatory properties capable of ameliorating insulin resistance. 

Among the individual *n*-3 PUFA, docosahexaenoic acid (DHA, C 22:6 *n*-3) concentration in maternal plasma positively associates with insulin sensitivity [29]. As a speculation, this insulin -ensitizing effect may be mediated through docosahexaenoic acid derivatives such as protectins, resolvins, and maresins. These anti-inflammatory molecules enhance hepatic β-oxidation, suppress lipogenesis, and stimulate adipokine secretion [30]. However, we cannot conclude whether the decreased serum concentration of *n*-3 PUFA is a cause or a consequence of the altered glucose–insulin axis.

We found an association between C-peptide and docosahexaenoic acid (DHA, C 22:6 *n*-3) in mothers bearing a female, but not when bearing a male, fetus. If confirmed in a larger cohort, this would add to the growing evidence of fetal sex influences on metabolism of the pregnant woman. Fetal sex through placental signals may modify maternal metabolic adaptations to pregnancy [9,23]. Interestingly, it has been shown that female placentas are more responsive to maternal dietary supplementation with *n*-3 PUFA [31].

Our study has several strengths. First, fatty acids were measured as concentrations rather than proportions (%) of fatty acids allowing us to identify changes in one fatty acid without being affected by that of others. Second, we covered a wide gestational age range within the first trimester (week 4^+0^–11^+6^). Third, smoking was objectively measured and smokers were excluded. This is important because of the known effect of smoking on fatty acids outside [32] and within pregnancy [33,34,35]. Some limitations deserve a comment. We do not have information on maternal diet, which is the sole source of essential fatty acids and the main source of long-chain PUFAs. However, if diet were to have an effect, then one would expect profound variation in the data for essential fatty acids, which was not observed here. Furthermore, the specific association between C-peptide and docosahexaenoic acid (DHA, C 22:6 *n*-3) in female-bearing mothers argues for differences in the metabolism of *n*-3 PUFAs rather than dietary habits. However, fetal sex was assessed in a small subgroup (female n = 46, male n = 37) and female-bearing mothers were over-represented in our cohort, which might limit the representativeness of the results. The cohort included fewer obese than overweight women, which may have affected the results, especially if the associations with BMI are not linear over the whole BMI range. We also lack information on maternal ethnicity, hence, the study needs replication in defined ethnic groups. 

## 4. Materials and Methods

### 4.1. Study Design and Participants

This was a secondary, prospective, cross-sectional study conducted in a non-academic setting between May 2017 and August 2018. The study was approved by the ethical committee of the Medical University of Graz (no. 29-095 ex 16/17, 23rd December 2016 and 31-094 ex 18/19, 1 March 2019). All participants provided written informed consent and all methods were performed in accordance with the relevant guidelines and regulations. The study included 123 pregnant women recruited during counseling for voluntary pregnancy termination (gestational age 4^+0^–11^+6^ weeks). Gestational age was defined as days post last menstrual period (LMP) and corroborated by ultrasound measurement of crown-rump length. Inclusion criteria were maternal age ≥ 18 years, gestational age < 12 weeks post LMP, and singleton pregnancy. Exclusion criteria were smoking, objectively assessed by serum cotinine measurements, and known co-morbidities, e.g., pre-existing diabetes mellitus, hypertension, auto-immune disease. Cohort characteristics are shown in Table 2.

### 4.2. Blood Collection and Storage

Venous blood (8 mL) was collected after overnight fasting. The serum fraction was separated by centrifugation (2000× *g* at 4 °C for 10 min) and immediately stored at −80 °C until further use, as previously described [19].

### 4.3. Maternal Metabolic Parameters

Smoking status was assessed by quantification of serum cotinine levels with a competitive immunoassay (Abnova, Taipei, Taiwan Cat# KA0930) using a cut-off of ≤0.03 nmol/L cotinine for non-smokers [36]. Analytical sensitivity of the assay was 2.5 nmol/L, cross-reactivities: nicotine < 1%, nicotinamide < 1%, nicotinic acid < 1%.

Maternal BMI was calculated as weight in kilogram divided by the square of height in meters (kg/m^2^), both measured at the time of blood collection, and used as an indicator of maternal overweight and obesity. Serum leptin (ng/mL) was measured by a sandwich immunoassay (DRG, Marburg, Germany, Cat# EIA2395) and used as a proxy for maternal fat mass. Intra-assay and inter-assay CVs were 6.2% and 6.6%, respectively. Analytical sensitivity was 0.7 ng/mL and recovery was 93.5% with no cross-reactivity with human insulin, proinsulin, C-peptide, glucagon, or IGF-I.

Fasting serum glucose (mmol/L) was measured using the hexokinase-based test (Glucose HK Gen.3, Roche Diagnostics, Mannheim, Germany) on an automated analyzer (cobas^®^ 8000 c701, Roche Diagnostics, Mannheim, Germany).

Fasting serum C-peptide (pmol/L) was measured by a Sandwich Immunoassay (R&D Systems, Minneapolis, MINN, USA Cat# DICP00). Intra-assay and inter-assay CVs were 3.1% and 8.3%, respectively. The analytical sensitivity was 2.88 pmol/L and recovery was 100.4%. Cross-reactivity of <0.5% was observed with recombinant human IGF I, IGF II, insulin, proinsulin, and relaxin.

Fasting serum glucose and fasting C-peptide concentrations were used to calculate the homeostatic model assessment of insulin sensitivity (IS_HOMA_) [37]:

$$ISHOMA=\;\frac{22.5}{Cpeptide\left(\frac{pmol}{l}\right)\;*glucose\;\left(\frac{mg}{dl}\right)}$$

### 4.4. Quantification of Serum Fatty Acids

Concentrations of serum fatty acids were quantified as previously described [38]. Briefly, serum lipids were extracted in chloroform:methanol (2:1) containing 0.005% (*w*/*v*) BHT and an internal standard of nonadecenoic acid (19:1). The final lipid extracts were evaporated to dryness under vacuum, resuspended in toluene, and subjected to methanolysis for 2.5 h at 80 °C in methanol:toluene (4:1) containing acetyl chloride and methyl-heptadecanoate (C 17:0) as a reference standard. The fatty acid methyl esters were separated and quantified on a Perkin Elmer gas chromatograph (Autosystem; Norwalk, CT, USA), with a flame ionization detector and a 30 m × 0.25 mm Omegawax capillary column. Nitrogen was used as carrier gas and the fatty acid methyl esters were compared with purified standards (Sigma Chemicals Co., St. Louis, MO, USA). Fatty acid concentrations (mg/L) were quantified as a function of the corresponding peak areas compared to that of the internal standard.

### 4.5. Fetal Sex Determination

For a subgroup of pregnancies (n = 83 women), placental tissue was available making sex determination possible by following a previously established method [39]. Briefly, placental tissue was homogenized in RLT Plus Buffer (Qiagen, Venlo, The Netherlands) with 1% β-Mercaptoethanol (*v*/*v*, Merck, Darmstat, Germany) in a tissue lyser (MagNa Lyser, Roche, Basel, Switzerland). RNA was isolated with the AllPrep DNA/RNA/miRNA Universal Kit (Qiagen, Hombrechtikon, Switzerland) according to the manufacturer’s instructions. Reverse transcription was performed using the LunaScript^TM^ RT SuperMix Kit (New England BioLabs, Frankfurt, Germany). TaqMan Universal PCR Master Mix (Life Technologies, Carlsbad, CA, USA) and the primers *DDX3Y* (FAM labeled) and *XIST* (VIC labeled) (Life Technologies, *DDX3Y*: Hs00965254_gH, *XIST*: Hs01079824_m1) were used for the RT-qPCR (CFX96 Thermocycler, BioRad Laboratories, Hercules, CA, USA). Cycle threshold (Ct) values were generated by the BioRad CFX Manager 3.1 software and fetal sex was determined based on the ΔCt (*XIST* Ct–*DDX3Y* Ct).

### 4.6. Exposures and Outcomes

Primary exposures were maternal obesity (BMI) and fat mass (leptin). Secondary exposures were fasting serum glucose, fasting serum C-peptide, and IS_HOMA_. Main outcomes were the individual fatty acids, total fatty acids, saturated fatty acids (SFA; myristic acid (C 14:0), palmitic acid (C 16:0), stearic acid (C 18:0)), monounsaturated fatty acids (MUFA; palmitoleic acid (C 16:1), oleic acid (C 18:1), erucic acid (C 22:1)), *n*-3 polyunsaturated fatty acids (*n*-3 PUFA; α-linolenic acid (C 18:3 *n*-3), eicosatrienoic acid (C 20:3 *n*-3), eicosapentaenoic acid (C 20:5 *n*-3), docosapentaenoic acid (C 22:5 *n*-3), docosahexaenoic acid (C 22:6 *n*-3)), and *n*-6 polyunsaturated fatty acids (*n*-6 PUFA; linoleic acid (C 18:2 *n*-6), y-linolenic acid (C 18:3 *n*-6), eicosadienoic acid (C 20:2 *n*-6), dihomo- y-linolenic acid (C 20:3 *n*-6), arachidonic acid (C 20:4 *n*-6), adrenic acid (C 22:4 *n*-6), and osbond acid (C 22:5 *n*-6)).

Indices of fatty acid desaturase activity were calculated as product/precursor ratios, i.e., palmitoleic acid (C16:1)/palmitic acid (C16:0) for Δ9-desaturase, ƴ-linolenic acid (C18:3 *n*-6)/linoleic acid (C18:2 *n*-6) for Δ6-desaturase, arachidonic acid (C20:4)/dihomo-y-linolenic acid (C20:3) for Δ5-desaturase, and arachidonic acid (C20:4 n6)/linoleic acid (18:2 *n*-6) for the sum of elongase, Δ5- and Δ6-desaturases activity [40,41,42].

### 4.7. Statistical Analyses

Normal distribution of variables was assessed visually with histograms and Q-Q plots and by comparison of mean and median values. Skewed data were ln-transformed prior to analysis. Normally distributed variables are presented as the mean ± SD and non-normally distributed variables as median and IQR.

Associations between metabolic parameters (BMI, leptin, glucose, C-peptide, and IS_HOMA_) and maternal serum fatty acids were explored using multivariate linear regression analysis. In the first step, the exposures were analyzed in a continuous and categorical model to test the assumption of linearity. In subsequent analyses, exposures were categorized, when the associations between exposures and outcomes were not linear (assessed by comparison of β estimates between dummy variables). Cut-off values of the categories were based on accepted criteria (under-/normal-weight: BMI < 24.9 kg/m^2^, overweight: BMI 25–29.9 kg/m^2^, obese: BMI ≥ 30 kg/m^2^) [43], or on tertiles, when there were no established cut-off points (leptin: leptin < 8.5 ng/mL, leptin 8.5–15.3 ng/mL, leptin ≥ 15.3 ng/mL; glucose: glucose < 4.28 mmol/L, glucose 4.28–4.97 mmol/L, glucose ≥ 4.97 mmol/L; C-peptide: C-peptide < 315.4 pmol/L, C-peptide 315.4–437.8 pmol/L, C-peptide ≥ 437.8 pmol/L; IS_HOMA_: IS_HOMA_ ≤ 0.59, IS_HOMA_ 0.58–0.85, IS_HOMA_ > 0.85) [19]. The most favorable category (1st tertile of leptin, glucose, and C-peptide 3rd tertile for IS_HOMA_) was used as reference.

To test for potential influences of gestational age on total fatty acids, SFA, MUFA, *n*-3, and *n*-6 PUFA concentrations, gestational age was added as a continuous and categorical variable to the models (gestational age: weeks 4–6, weeks 7–9, weeks 10–12). Based on β-estimates, the associations with gestational age were linear and, therefore, gestational age was further used as a continuous covariate. Statistical interactions between gestational age and BMI, leptin, glucose, C-peptide, and IS_HOMA_, respectively, were not significant (for all: *p* > 0.1).

In a first model, the a priori defined covariates gestational age (days), maternal age (years), and processing time (minutes) were included. In a second model, BMI was added as a covariate to examine a potential confounding effect in the associations between fatty acids and glucose, C-peptide, and IS_HOMA_. Leptin replaced BMI in a third model.

The Mann–Whitney U test was used to assess the representativeness of the sub-group (n = 83) for which fetal sex data was available with respect to the total cohort (n = 123). The Mann–Whitney U test was also used to compare maternal metabolic parameters between mothers bearing a female or a male fetus. Interactions between fetal sex and BMI, leptin, glucose, C-peptide, and IS_HOMA_, respectively, were tested by including both variables and all covariates in the regression model and an interaction term. If the interaction term was significant (*p* < 0.1), associations between exposures (BMI, leptin, glucose, C-peptide, and IS_HOMA_) and outcomes (fatty acids) were examined in the female-bearing or male-bearing subgroups separately, using the above-described linear regression models.

A *p*-value < 0.05 was considered statistically significant, except for interaction terms, where a *p*-value *p* < 0.1 was considered significant. Furthermore, for the associations with fatty acids, the Benjamini–Hochberg procedure was applied to correct for multiple testing (FDR = 0.2), and in the results, it is always indicated whether a *p*-value was below the critical value for statistical significance.

Data analyses were conducted in IBM SPSS statistics (version 25, IBM Corp, Armonk, NY, USA) and graphs were produced using GraphPad Prism (version 8.4.2, GraphPad Software, San Diego, CA, USA).

## 5. Conclusions

In conclusion, maternal C-peptide and insulin sensitivity (IS_HOMA_), but not proxies of obesity such as BMI or leptin, associate with serum *n*-3 PUFA. Pregnant women (week 4^+0^–11^+6^) with a high C-peptide concentration and low insulin sensitivity (IS_HOMA_) had decreased serum concentration of total *n*-3 PUFA and docosahexaenoic acid (DHA, C 22:6 *n*-3). Furthermore, we found an association between C-peptide and docosahexaenoic (DHA, C 22:6 *n*-3) acid in female-bearing mothers, but not in male-bearing mothers. If confirmed in a larger cohort, these results add to the growing evidence that fetal sex influences metabolism in pregnant women.

## Figures and Tables

**Table 1:** Associations between C-peptide or IS_HOMA_ and fatty acids classes are independent of BMI and leptin.

		Model 1			Model 2			Model 3		
		*β*	*CI*	*p*	*β*	*CI*	*p*	*β*	*CI*	*p*
C-peptide^a^	Total fatty acids	−14.9	−201.4–171.7	0.875	−7.5	−202.1–187.1	0.939	−43.3	−243.0–156.5	0.669
	SFA	0.6	−66.0–67.3	0.985	6.5	−62.9–75.9	0.853	−0.7	−72.3–70.8	0.984
	MUFA	19.0	−38.3–76.3	0.512	21.2	−38.5–81.0	0.483	8.5	−52.7–69.8	0.783
	PUFA *n*-3	**−17.0**	**−31.2–−2.8**	**0.019**	**−17.1**	**−32.0–−2.3**	**0.024**	−21.5	**−36.5–−6.4**	**0.006**
	PUFA *n*-6	−17.5	−93.25–−58.3	0.648	−18.1	−97.1–61.0	0.652	−29.7	−110.8–−51.4	0.470
	Ratio *n*-6/*n*-3	**0.69**	**0.12–1.25**	**0.018**	**0.69**	**0.10–1.28**	**0.022**	0.78	**0.17–1.38**	**0.012**
IS_HOMA_^a^	Total fatty acids	35.0	−131.2–201.3	0.677	30.9	−142.1–203.8	0.677	69.6	−111.4–250.5	0.448
	SFA	10.5	−48.9–69.9	0.727	6.5	−55.2–68.1	0.836	14.4	−50.4–79.2	0.661
	MUFA	−8.5	−59.7–42.6	0.742	−9.1	−62.3–44.1	0.735	3.3	−52.3– 58.8	0.908
	PUFA *n*-3	**15.1**	**2.5–27.8**	**0.020**	**15.2**	**2.0–28.3**	**0.025**	20.1	**6.4–33.7**	**0.004**
	PUFA *n*-6	18.0	−49.6–85.5	0.599	18.4	−51.9–88.6	0.605	31.8	−41.6–105.3	0.392
	Ratio *n*-6/*n*-3	**−0.59**	**−1.09–−0.09**	**0.022**	**−0–0.59**	**−1.12–−0.07**	**0.027**	**−0.69**	**−1.24–−1.14**	**0.014**

Model 1: adjusted for gestational age, maternal age, and processing time. Model 2: Model 1 + BMI adjustment. Model 3: Model 1 + leptin adjustment. Bold: Statistically significant difference after correction for multiple testing (FDR = 0.2). β: β estimate; CI: 95% confidence interval. SFA: sum of 14:0, 16:0, and 18:0; MUFA: sum of 16:1, 18:1, and 22:1; *n*-3 PUFA: sum of 18:3, 20:3, 20:5, 22:5, and 22:6; *n*-6 PUFA: sum of 18:2, 18:3, 20:3, 20:4, 22:2, 22:4, and 22:5. An ln-transformed variable. Non-normally distributed variables were ln-transformed to meet the requirements of multivariate linear regression analysis. Each 1% increase in the predictor variable (C-peptide and ISHOMA) increases the outcome variable by 1/100 units of the estimate. For example, a 1% increase in C-peptide is associated with a 0.17 mg/L decrease in *n*-3 PUFA.

**Table 2:** Characteristics of study participants (N = 123).

	n (%)	Mean ± SD	Median (IQR)
Age (years)	122	31.4 (± 7.2)	
Gestational age (days)	123	51.0 (± 15.4)	
4–6 weeks	58 (47.0%)		35.0 (35.0–42.0)
7–9 weeks	48 (39.0%)		56.0 (51.0–61.0)
10–12 weeks	17 (13.8%)		81.0 (78.0–82.0)
BMI (kg/m^2^)	123		22.6 (20.6–25.7)
Under-/normal-weight (<25 kg/m^2^)	85 (69.2%)		21.1 (19.8–22.8)
Overweight (25.0–29.9 kg/m^2^)	30 (24.4%)		26.9 (25.9–27.4)
Obese (≥30.0 kg/m^2^)	8 (6.5%)		32.6 (31.9–39.5)
Metabolic parameters			
Leptin (ng/mL)	123		11.8 (7.1–17.4)
1st tertile (<8.5)	41		5.4 (3.5–7.1)
2nd tertile (8.5–15.3)	41		12.0 (10.2–13.9)
3rd tertile (≥15.3)	41		19.6 (17.4–28.6)
Glucose (mmol/L)	118	4.76 (± 0.85)	
1st tertile (<4.28)	39		4.01 (3.65–4.14)
2nd tertile (4.28–4.97)	40		4.65 (4.49–4.82)
3rd tertile (≥4.97)	39		5.52 (5.16–5.98)
C-peptide (pmol/L)	123		371.0 (281.5–484.9)
1st tertile (<315.4)	41		258.3 (218.5–281.7)
2nd tertile (315.4–437.8)	41		371.0 (346.6–402.8)
3rd tertile (≥437.8)	41		537.0 (476.4–621.3)
IS_HOMA_	118		0.74 (0.53–1.00)
1st tertile (≤0.59)	40		0.46 (0.36–0.53)
2nd tertile (0.58–0.85)	39		0.73 (0.68–0.79)
3rd tertile (>0.85)	39		1.13 (1.00–1.41)
Fatty acids (mg/L)			
Total fatty acids	123	2724.2 (± 415.4)	
SFA	123	918.9 (± 148.2)	
C 14:0	123		22.7 (17.3–32.8)
C 16:0	123		618.3 (539.4–694.0)
C 18:0	123	273.6 (± 45.5)	
MUFA	123	610.5 (± 132.8)	
C 16:1	123		54.8 (39.7–67.8)
C 18:1 (*n*-9)	123	546.1 (± 114.2)	
C 22:1 (*n*-9)	123	6.5 (± 2.8)	
*n*-3 PUFA	123	158.0 (± 28.5)	
C 18:3 (*n*-3)	123		10.7 (8.7–14.8)
C 20:3 (*n*-3)	123		4.9 (3.6–7.3)
C 20:5 (*n*-3)	123	17.2 (± 7.3)	
C 22:5 (*n*-3)	123	26.6 (± 9.6)	
C 22:6 (*n*-3)	123	97.5 (± 24.1)	
*n*-6 PUFA	123	1036.9 (± 161.8)	
C 18:2 (*n*-6)	123	751.4 (± 131.9)	
C 18:3 (*n*-6)	123		9.7 (7.8–12.9)
C 20:2 (*n*-6)	123		6.2 (5.0–7.3)
C 20:3 (*n*-6)	123	45.4 (± 14.0)	
C 20:4 (*n*-6)	123	216.4 (± 49.7)	
C 22:4 (*n*-6)	123	7.4 (± 2.4)	
C 22:5 (*n*-6)	123	5.8 (± 4.0)	

Normally distributed variables are presented as the mean ± SD and those not normally distributed as median (IQR). BMI: Body mass index; gestational age: postmenstrual period; IS_HOMA_: Homeostatic model assessment of insulin sensitivity; SD: standard deviation; IQR: Interquartile range.

## Data Availability

The data presented in this study are available on request from the corresponding author. The data are not publicly available due to privacy/ethical restrictions.

## Supplementary Materials

The following are available online at https://www.mdpi.com/article/10.3390/ijms221910422/s1.
