# Supplementary material for: Maternal C-Peptide and Insulin Sensitivity, but Not BMI, Associate with Fatty Acids in the First Trimester of Pregnancy
Source: Int J Mol Sci. 2021 Sep 27;22(19):10422. doi: 10.3390/ijms221910422 (PMC8508886; doi:10.3390/ijms221910422)

Table S1. Associations between individual BMI, leptin, glucose, C-peptide and IS_{HOMA} and SFA (A), MUFA (B), *n*-3 PUFA (C) and *n*-6 PUFA (D).

A) SFA

	**C 14:0 [a]**			**C 16:0 [a]**			**C 18:0**		
Predictors	*β*	*CI*	*p*	*β*	*CI*	*p*	*β*	CI	*p*
BMI < 25 *kg/m²*	ref			ref			ref		
BMI 25-29.9 *kg/m²*	**-0.20**	**-0.39 – -0.01**	**0.037**	-0.06	-0.14 – 0.03	0.200	13.45	-4.69 – 31.69	0.144
BMI ≥ 30 *kg/m²*	-0.11	-0.45 – 0.21	0.488	-0.05	-0.16 – 0.10	0.520	-0.61	-32.10 – 30.88	0.969
Leptin < 8.5 *ng/ml*	ref			ref			ref		
Leptin 8.5-15.3 *ng/ml*	-0.07	-013 – 0.27	0.496	-0.02	-0.11 – 0.07	0.704	-8.09	-27.57 – 11.40	0.413
Leptin ≥ 15.3 *ng/ml*	-0.51	-0.15 – 0.26	0.617	-0.02	-0.11 – - 0.08	0.747	-109	-20.70 – 18.52	0.913
Glucose < 4.28 *mmol/l*	ref			ref			ref		
Glucose 4.28-4.97 *mmol/l*	0.06	-0.14 – 0.26	0.530	-0.01	-0.10 – 0.08	0.772	3.17	-16.12 – 22.46	0.745
Glucose ≥ 4.97 *mmol/l*	-0.15	-0.38 – 0.09	0.220	**-0.11**	**-0.22 – -0.01**	**0.032**	-10.60	-33.23 – 12.03	0.355
C-peptide < 315.4 *pmol/l*	ref			ref			ref		
C-peptide 315.4–437.8 *pmol/l*	0.17	-0.03 – 0.37	0.089	0.03	-0.06 – 0.12	0.572	-4.23	-23.62 – 15.15	0.666
C-peptide ≥ 437.8 *pmol/l*	0.04	-0.16 – 0.25	0.675	-0.05	-0.14 – 0.05	0.314	-20.08	-21.84 –17.83	0.841
IS_{HOMA} ≤ 0.59	-0.05	-0.26 – 0.17	0.664	-0.10	-0.19 – 0.00	0.053	-10.68	-31.46 – 10.10	0.311
IS_{HOMA} 0.58-0.85	-0.05	-0.26 – 0.16	0.643	-0.02	-0.11 – 0.08	0.710	-5.70	-26.06 – 14.67	0.580
IS_{HOMA} > 0.85	ref			ref			ref		

B) MUFA

	C 16:1 [a]			C 18:1 (*n*-9)			C 22:1 (*n*-9)		
Predictors	*β*	*CI*	*p*	*β*	CI	*p*	*β*	*CI*	*p*
BMI < 25 *kg/m²*	ref			ref			ref		
BMI 25-29.9 *kg/m²*	0.01	-0.16 – 0.19	0.932	-28.40	-75.47 – 18.67	0.235	-0.71	-1.90 – 0.48	0.239
BMI ≥ 30 *kg/m²*	0.14	-0.18 – 0.46	0.379	25.31	-56.17 – 106.78	0.540	0.86	-1.20 – 2.92	0.411
Leptin < 8.5 *ng/ml*	ref			ref			ref		
Leptin 8.5-15.3 *ng/ml*	0.11	-0.09 – 0.30	0.268	-1.90	-52.29 – 48.49	0.941	0.17	-1.11 – 1.44	0.798
Leptin ≥ 15.3 *ng/ml*	0.16	-0.04 – 0.35	0.114	17.25	-33.47 – 67.97	0.502	0.28	-1.01 – 1.57	0.667
Glucose < 4.28 *mmol/l*	ref			ref			ref		
Glucose 4.28-4.97 *mmol/l*	0.11	-0.08 – 0.30	0.256	-18.73	-68.64 – 31.18	0.459	-0.68	-1.93 – 0.57	0.283
Glucose ≥ 4.97 *mmoll/l*	-0.17	-0.40 – 0.05	0.126	-43.60	-102.16 – 14.95	0.143	0.89	-0.57 – 2.36	0.229
C-peptide < 315.4 *pmol/l*	ref			ref			ref		
C-peptide 315.4–437.8 *pmol/l*	0.19	-0.01 – 0.38	0.053	18.32	-31.48 – 68.13	0.468	-0.45	-1.71 – 0.82	0.486
C-peptide ≥ 437.8 *pmol/l*	0.05	-0.15 – 0.25	0.610	-14.31	-65.27 – 36.65	0.579	-0.32	-1.62 – 0.97	0.622
IS_{HOMA} ≤ 0.59	-0.06	-0.27 – 0.15	0.594	-40.13	-93.90 – 13.64	0.142	-0.21	-1.58 – 1.17	0.768
IS_{HOMA} 0.58-0.85	0.00	-0.21 – 0.20	0.967	-16.39	-69.09 – 36.30	0.539	-0.79	-2.13 – 0.56	0.249
IS_{HOMA} > 0.85	ref			ref			ref		

C) *n*-3 PUFA

	C 18:3 (*n*-3) [a]			C 20:3 (*n*-3) [a]			C 20:5 (*n*-3)		
Predictors	*β*	*CI*	*p*	*β*	*CI*	*p*	*β*	*CI*	*p*
BMI < 25 kg/m^2	ref			ref			ref		
BMI 25-29.9 kg/m^2	-0.09	-0.25 – 0.07	0.291	0.20	-0.06 – 0.46	0.128	0.25	-2.79 – 3.29	0.869
BMI ≥ 30 kg/m^2	0.04	-0.24 – 0.31	0.794	-0.11	-0.54 – 0.34	0.638	-0.10	-5.36 – 5.17	0.971
Leptin < 8.5 *ng/ml*	ref			ref			ref		
Leptin 8.5-15.3 *ng/ml*	0.02	-0.15 – 0.20	0.778	0.04	-0.24 – 0.31	0.779	-0.56	-3.78 – 2.65	0.729
Leptin ≥ 15.3 *ng/ml*	0.08	-0.09 – 0.26	0.339	0.19	-0.08 – 0.47	0.167	-2.12	-5.35 – 1.12	0.197
Glucose < 4.28 *mmol/l*	ref			ref			ref		
Glucose 4.28-4.97 *mmol/l*	0.01	-0-16 – 0.18	0.916	-0.10	-0.36 – 0.19	0.520	- 0.458	-3.67 – 2.75	0.778
Glucose ≥ 4.97 *mmoll/l*	0.02	-0.18 – 0.22	0.842	-0.20	-0.34 – 0.30	0.901	1,121	-2.65 – 4.89	0.557
C-peptide < 315.4 *pmol/l*	ref			ref			ref		
C-peptide 315.4–437.8 *pmol/l*	0.05	-0.12 – 0.22	0.569	**-0.46**	**-0.71 – -0.19**	**0.001***	-2.48	-5.63 – 0.67	0.122
C-peptide ≥ 437.8 *pmol/l*	-0.03	-0.20 – 0.15	0.757	**-0.36**	**-0.62 – -0.10**	**0.008**	**-3.33**	**-6.55 – -0.10**	**0.043**
IS_{HOMA} ≤ 0.59	-0.06	-0.24 - -0.12	0.521	-0.21	-0.50 – 0.08	0.153	-3.24	-6.66 – 0.19	0.064
IS_{HOMA} 0.58-0.85	-0.10	-0.27 – 0.08	0.285	-0.24	-0.52 – 0.05	0.101	-1.87	-5.23 – 1.48	0.270
IS_{HOMA} > 0.85	ref			ref			ref		

Predictors	C 22:5 (*n*-3) *β*	*CI*	*p*	C 22:6 (*n*-3) *β*	*CI*	*p*
BMI < 25 *kg/m²*	ref			ref		
BMI 25-29.9 *kg/m²*	0.24	-3.91 – 4.39	0.908	6.37	-3.77 – 16.50	0.216
BMI ≥ 30 *kg/m²*	-4.50	-11.68 – 2.68	0.217	-3,075	-20.62 – 14.47	0.729
Leptin < 8.5 *ng/ml*	ref			ref		
Leptin 8.5-15.3 *ng/ml*	2.97	-1.43 – 7.37	0.183	1.73	-9.14 – 12.60	0.753
Leptin ≥ 15.3 *ng/ml*	0.09	-4.34 – 4.52	0.969	1.12	-9.83 – 12.06	0.840
Glucose < 4.28 *mmol/l*	ref			ref		
Glucose 4.28-4.97 *mmol/l*	1.31	3.12 – 5.73	0.560	-8,152	-18.90 – 2.59	0.136
Glucose ≥ 4.97 *mmoll/l*	-0.89	-6.08 – 4.30	0.734	-3,513	-16.12 – 9.09	0.582
C-peptide < 315.4 *pmol/l*	ref			ref		
C-peptide 315.4–437.8 *pmol/l*	-3.51	-7.83 – 0.80	0.109	-3.57	-14.21 – 7.07	0.507
C-peptide ≥ 437.8 *pmol/l*	**-4.93**	**-9.34 – -0.52**	**0.029**	-9.88	-20.77 – 1.00	0.075
IS_{HOMA} ≤ 0.59	**-5.37**	**-10.08 - -0.67**	**0.26**	-10.37	-21.96 – 1.22	0.079
IS_{HOMA} 0.58-0.85	-2.93	-7.54 – 1.68	0.210	-5.27	-16.62 – 6.09	0.360
IS_{HOMA} >0.85	ref			ref		

D) *n*-6 PUFA

	**C 18:2 (*n*-6)**			**C 18:3 (*n*-6) [a]**			**C 20:2 (*n*-6) [a]**			**C 20:3 (*n*-6)**		
Predictors	*β*	CI	*p*	*β*	CI	*p*	*β*	*CI*	*p*	*β*	*CI*	*p*
BMI < 25 *kg/m²*	ref			ref			ref			ref		
BMI 25-29.9 *kg/m²*	-31.96	-88.61 – 24.70	0.266	-0.08	-0.28 – 0.11	0.395	0.11	-0.05 – 0.26	0.177	4.27	-1.4 – 9.93	0.138
BMI ≥ 30 *kg/m²*	-40.28	-138.34 – 57.79	0.418	-0.05	-0.39 – 0.29	0.765	0.07	-0.20 – 0.34	0.604	4.34	-5.47 – 14.14	0.383
Leptin < 8.5 *ng/ml*	ref			ref			ref			ref		
Leptin 8.5-15.3 *ng/ml*	-23.98	-84.55 – 36.58	0.434	0.16	-0.05 – 0.37	0.126	-0.03	-0.20 – 0.14	0.720	3.90	-2.9 – 9.08	0.309
Leptin ≥ 15.3 *ng/ml*	-61.06	-67.07 – 54.85	0.843	0.10	-0.11 – 0.30	0.355	-0.04	-0.20 – 0.13	0.672	**6.22**	**0.19 – 12.25**	**0.043**
Glucose < 4.28 *mmol/l*	ref			ref			ref			ref		
Glucose 4.28-4.97 *mmol/l*	-2.467	-63.07 – 58.14	0.936	0.17	-0.03 – 0.37	0.095	**-0.20**	**-0.36 – -0.04**	**0.015**	-3.04	-9.08 – 3.00	0.321
Glucose ≥ 4.97 *mmoll/l*	-14.32	-85.42 – 56.78	0.691	-0.01	-0.25 – 0.22	0.920	**-0.23**	**-0.42 – -0.045**	**0.016**	-4.09	-11.17 – 2.99	0.255
C-peptide < 315.4 *pmol/l*	ref			ref			ref			ref		
C-peptide 315.4–437.8 *pmol/l*	-57.90	-65.65 – 54.07	0.848	-0.08	-0.29 – 0.12	0.428	-0.11	-0.27 – 0.05	0.187	0.05	-6 – 6.10	0.988
C-peptide ≥ 437.8 *pmol/l*	-36.56	-97.81 – 24.69	0.239	0.01	-0.20 – 0.22	0.920	**-0.18**	**-0.35 – -0.02**	**0.031**	0.8	-5.39 – 7	0.798
IS_{HOMA} ≤ 0.59	-49.34	-113.58 – 14.90	0.131	-0.12	-0.34 – 0.10	0.296	-0.16	-0.33 – 0.02	0.085	-0.31	-6.90 – 6.27	0.925
IS_{HOMA} 0.58-0.85	11.10	-51.85 – 74.06	0.727	-0.08	-0.29 – 0.14	0.472	-0.08	-0.25 – 0.10	0.394	-0.23	-6.68 – 6.22	0.944
IS_{HOMA} ≤ 0.85	ref			ref			ref			ref		

Predictors	C 20:4 (*n*-6) *β*	*CI*	*p*	C 22:4 (*n*-6) *β*	*CI*	*p*	C 22:5 (*n*-6) *β*	*CI*	*p*
BMI < 25 *kg/m²*	ref			ref			ref		
BMI 25-29.9 *kg/m²*	16.50	-4.16 – 37.17	0.116	0.93	-0.04 – 1.89	0.060	0.00	-1.67 – 1.67	0.997
BMI ≥ 30 *kg/m²*	20.20	-15.57 – 55.97	0.266	0.75	-0.93 – 2.42	0.379	-1.04	-3.93 – 1.85	0.479
Leptin < 8.5 *ng/ml*	ref			ref			ref		
Leptin 8.5-15.3 *ng/ml*	-2.97	-25.10 – 19.15	0.790	-0.02	-1.06 – 1.03	0.975	0.88	-0.89 – 2.65	0.328
Leptin ≥ 15.3 *ng/ml*	11.54	-10.73 – 33.81	0.307	0.33	-0.72 – 1.38	0.540	0.05	-1.73 – 1.83	0.956
Glucose < 4.28 *mmol/l*	ref			ref			ref		
Glucose 4.28-4.97 *mmol/l*	-1.01	-23.16 – 21.14	0.928	-0.44	-1.44 – 0.56	0.382	-0.87	-2.63 – 0.88	0.326
Glucose ≥ 4.97 *mmoll/l*	-9.13	-35.12 – 16.86	0.488	**-1.84**	**-3.01 – -0.67**	**0.002***	-1.78	-3.84 – 0.27	0.088
C-peptide < 315.4 *pmol/l*	ref			ref			ref		
C-peptide 315.4–437.8 *pmol/l*	-8.25	-30.31 – 13.81	0.461	-0.12	-1.16 – 0.91	0.815	-1.70	-3.44 – 0.04	0.055
C-peptide ≥ 437.8 *pmol/l*	1.10	-21.48 – 23.67	0.924	0.05	-1.01 – 1.11	0.929	-1.29	-3.07 – 0.49	0.153
IS_{HOMA} ≤ 0.59	-3.13	-27.02 – 20.75	0.464	-0.39	-1.51 – 0.72	0.487	-1.56	-3.46 – -0.34	0.107
IS_{HOMA} 0.58-0.85	8.69	-14.72 – 32.09	0.464	0.45	-0.64 – 1.54	0.418	-1.38	-3.24 – 0.48	0.144
IS_{HOMA} > 0.85	ref			ref			ref		

BMI: Body mass index; IS_{HOMA}: Homeostatic model assessment of insulin sensitivity; SFA: Saturated fatty acids; MUFA: Monounsaturated fatty acids. PUFA: polyunsaturated fatty acids. *β*: coefficient estimate; CI: Confidence interval; ref: reference category. Adjusted for: gestational age (days), maternal age (years) and processing time (minutes). [a] ln-transformed variable. Non-normally distributed variables were ln-transformed to meet the requirements of multivariate linear regression analysis. When the outcome variable (i.e. fatty acid) is ln-transformed, the *β* estimate needs to be exponentiated and substracted by 1. Bold font indicates p < 0.05. * Statistically significant after correcting for multiple testing using Benjamin Hochberg procedure (FDR=0.2).

Table S2. Comparison of maternal characteristics, metabolic parameters and concentrations of fatty acid classes between total cohort and subcohort, in which fetal sex was determined

	Total cohort n=123		Subpopulation n=83		
	**Mean ± SD**	**Median (IQR)**	**Mean ± SD**	**Median (IQR)**	***p*-value**
Age (*years*)	31.4 (± 7.2)		30.6 (± 7.5)		NS
Gestational age (*days*)	51.0 (± 15.4)		52.9 (± 15.6)		NS
BMI (*kg/m^2*)		22.6 (21.0 - 24.6)		22.9 (21.5 - 24.9)	NS
**Metabolic parameters**					
Leptin (*ng/ml*)		11.8 (8.5 - 15.3)		12.8 (8.7 - 16.0)	NS
Glucose (*mmol/l*)	4.76 (± 0.85)		4.68 (± 0.74)		NS
C-peptide (pmol/l)		371.0 (315.4 - 437.8)		376.8 (310.6 - 440.0)	NS
IS_{HOMA}		0.74 (0.59 - 0.85)		0.74 (0.59 - 0.86)	NS
Total fatty acids (*mg/l*)	2737.0 ± 436.8		2724.0 ± 415.4		NS
SFA (*mg/l*	921.6 ± 158.0		918.9 ± 148.2		NS
MUFA (*mg/l*)	611.6 ± 134.7		610.5 ± 132.8		NS
n-3 PUFA (*mg/l*)	159.2 ± 33.1		158.0 ± 28.5		NS
n-6 PUFA (*mg/l*	1044.6 ± 173.2		1036.9 ± 161.8		NS

BMI: Body mass index; IS_{HOMA}: Homeostatic model assessment of insulin sensitivity; SFA: Saturated fatty acids; MUFA: Monounsaturated fatty acids. PUFA: polyunsaturated fatty acids. SD: standard deviation; IQR: Interquartile range. Mann-Whitney U Test. NS: Not-statistically significant ($p > 0.05$).

Table S3. Sensitivity analysis, excluding data of women with glucose values > 7 mmol/l. Associations between individual BMI, leptin, glucose, C-peptide and IS_{HOMA} and SFA (A), MUFA (B), *n*-3 PUFA (C) and *n*-6 PUFA (D).

A) SFA

	**C 14:0 [a]**			**C 16:0 [a]**			**C 18:0**		
Predictors	*β*	*CI*	*p*	*β*	*CI*	*p*	*β*	CI	*p*
Glucose < 4.28 *mmol/l*	ref			ref			ref		
Glucose 4.28-4.97 *mmol/l*	0.07	-0.13 – 0.27	0.516	-0.01	-0.10 – 0.08	0.783	3.37	-15.88 – 22.62	0.730
Glucose ≥ 4.97 *mmol/l*	-0.15	-0.39 – 0.09	0.207	**-0.12**	**-0.22** – **-0.01**	**0.033**	-10.07	-32.73 – 12.60	0.381

B) MUFA

	**C 16:1 [a]**			**C 18:1 (*n*-9)**			**C 22:1 (*n*-9)**		
Predictors	*β*	*CI*	*p*	*β*	CI	*p*	*β*	*CI*	*p*
Glucose < 4.28 *mmol/l*	ref			ref			ref		
Glucose 4.28-4.97 *mmol/l*	0.11	-0.08 – 0.30	0.254	-18.67	-69.05 – 31.71	0.464	-0.69	-1.94 – 0.57	0.280
Glucose ≥ 4.97 *mmoll/l*	-0.18	-0.40 – 0.05	0.125	-43.80	-103.11 – 15.52	0.146	0.94	-0.54 – 2.42	0.210

C) n-3 PUFA

	**C 18:3 (*n*-3)** [a]			**C 20:3 (*n*-3)** [a]			**C 20:5 (*n*-3)**		
Predictors	*β*	*CI*	*p*	*β*	*CI*	*p*	*β*	*CI*	*p*
Glucose < 4.28 *mmol/l*	ref			ref			ref		
Glucose 4.28-4.97 *mmol/l*	0.01	-0-16 – 0.18	0.898	-0.09	-0.37 – 0.19	0.519	-0.47	-3.68 – 2.75	0.775
Glucose ≥ 4.97 *mmoll/l*	0.01	-0.19 – 0.21	0.945	-0.02	-0.34 – 0.31	0.913	1,35	-2.44 – 5.13	0.482

	**C 22:5 (*n*-3)**			**C 22:6 (*n*-3)**		
Predictors	*β*	*CI*	*p*	*β*	*CI*	*p*
Glucose < 4.28 *mmol/l*	ref			ref		
Glucose 4.28-4.97 *mmol/l*	-1.31	-3.15 – 5.78	0.561	-8.25	-19.04– 2.54	0.133
Glucose ≥ 4.97 *mmoll/l*	-0.83	-6.08 – 4.42	0.755	-3.04	-15.75 – 9.67	0.636

D) n-6 PUFA

Predictors	C 18:2 (*n*-6)			C 18:3 (*n*-6) [a]			C 20:2 (*n*-6) [a]			C 20:3 (*n*-6)		
	β	CI	*p*	*β*	CI	*p*	*β*	*CI*	*p*	*β*	*CI*	*p*
Glucose < 4.28 *mmol/l*	ref			ref			ref			ref		
Glucose 4.28-4.97 *mmol/l*	-2.17	-63.25 – 59.91	0.944	0.18	-0.06 – 0.36	0.058	**-0.20**	**-0.36 – -0.04**	**0.015**	-3.04	-9.10 – 3.05	0.326
Glucose ≥ 4.97 *mmoll/l*	-15.90	-87.82 – 56.02	0.662	-0.01	-0.23 – 0.21	0.924	**-0.25**	**-0.43 – -0.06**	**0.011**	-3.09	-11.06 – 3.25	0.282

Predictors	C 20:4 (*n*-6)			C 22:4 (*n*-6)			C 22:5 (*n*-6)		
	β	*CI*	*p*	*β*	*CI*	*p*	*β*	*CI*	*p*
Glucose < 4.28 *mmol/l*	ref			ref			ref		
Glucose 4.28-4.97 *mmol/l*	-1.06	-23.27 – 21.16	0.925	-0.45	-1.45 – 0.56	0.382	-0.86	-2.63 – 0.90	0.335
Glucose ≥ 4.97 *mmoll/l*	-7.81	-33.97 – 18.34	0.555	**-1.82**	**-3.00– -0.63**	**0.003**	-1.80	-3.88 – 0.28	0.088

BMI: Body mass index; IS_{HOMA}: Homeostatic model assessment of insulin sensitivity; SFA: Saturated fatty acids; MUFA: Monounsaturated fatty acids. PUFA: polyunsaturated fatty acids. *β*: coefficient estimate; CI: Confidence interval; ref: reference category. Adjusted for: gestational age (days), maternal age (years) and processing time (minutes). [a] ln-transformed variable. Non-normally distributed variables were ln-transformed to meet the requirements of multivariate linear regression analysis. When the outcome variable (i.e. fatty acid) is ln-transformed, the *β* estimate needs to be exponentiated and substracted by 1. Bold font indicates $p < 0.05$. The associations lost significance after correcting for multiple testing using Benjamin Hochberg procedure (FDR=0.2).

Table S4. Comparison of maternal characteristics, metabolic parameters and concentrations of fatty acid classes between mothers bearing female and male fetuses.

	Mothers bearing female fetuses n=46		Mothers bearing male fetuses n=37		
	**Mean ± SD**	**Median (IQR)**	**Mean ± SD**	**Median (IRQR)**	***p*-value**
Age (*years*)	32 (± 7.3)		29 (± 7.4)		NS
Gestational age (*days*)	51.5 (± 15.5)		54.6 (± 15.7)		NS
BMI (kg/m^2)		**23.6 (22.7 – 25.9)**		**22.0 (21.0 – 23.1)**	**<0.05**
**Metabolic parameters**					
Leptin (*ng/ml*)		15.2 (11.6 – 17.5)		11.5 (8.4 - 14.3)	NS
Glucose (*mmol/l*)	4.66 (± 0.72)		4.73 (± 0.75)		NS
C-peptide (*pmol/l*)		402.3 (316.0 – 445.7)		398.6 (293.4 – 400.4)	NS
IS_{HOMA}		0.69 (0.57 - 0.90)		0.81 (0.67 - 0.87)	NS
Total fatty acids (*mg/l*)	2704.9 ± 458.8		2748.2 ± 359.0		NS
SFA (*mg/l*)	915.2 ± 160.5		923.4 ± 133.3		NS
MUFA (*mg/l*)	606.1 ± 138.6		615.9 ± 126.9		NS
n-3 PUFA (*mg/l*)	158.8 ± 31.9		157.1± 24.0		NS
n-6 PUFA (*mg/l*)	1024.7 ± 174.6		1051.9 ± 145.2		NS

BMI: Body mass index; IS_{HOMA}: Homeostatic model assessment of insulin sensitivity; SFA: Saturated fatty acids; MUFA: Monounsaturated fatty acids. PUFA: polyunsaturated fatty acids. SD: standard deviation; IQR: Interquartile range. Mann-Whitney U Test. NS: Non-statistically significant ($p > 0.05$). Statistically significant results in bold. Note: Although the concentration of individual fatty acids, TFA, SFA, MUFA and *n*-3 and *n*-6 PUFA did not significantly differ between female and male bearing mothers, there was a significant interaction ($p < 0.1$) between fetal sex and C-peptide, fetal sex and IS_{HOMA} and fetal sex and BMI in the model for *n*-3 PUFA (adjusted for processing time, maternal age and gestational age).

Table S5. Effect size of the association between C-peptide and docosahexaenoic acid (DHA) in the female and male subgroups.

	**Female-bearing mothers (n=46)**		**Male-bearing mothers (n=37)**	
	Effect-size	*p*-value	Effect-size	*p*-value
**Model 1**	23.3%	<0.05	13.5%	NS
**Model 2**	24.3%	NS	13.5%	NS
**Model 3**	24.1%	NS	15.4%	NS

Model 1: Adjusted for gestational age, maternal age and processing time

Model 2: Model 1 + Adjustment for BMI

Model 3: Model 1 + Adjustment for leptin

NS: Not statistically significant ($p > 0.05$);

Figure S1. Association of fasting glucose with individual SFA. Measures of maternal fasting glucose were categorized into tertiles and the estimates compared to the referent category: glucose < 4.28 mmol/l. Model adjusted for gestational age (days), maternal age (years) and processing time (minutes). * $p < 0.05$. The association lost significance after correcting for multiple testing using Benjamin Hochberg procedure (FDR=0.2) (see **Table S1**).

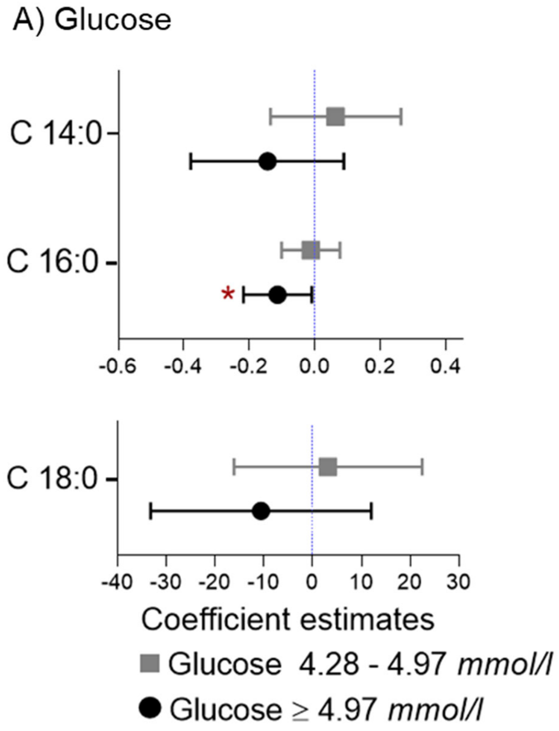

Figure S2. Sensitivity analysis: full cohort excluding women with glucose values > 7 mmol/l. Measures of maternal fasting glucose were categorized into tertiles and the estimates compared to the referent category: glucose < 4.28 mmol/l. Model adjusted for gestational age (days), maternal age (years) and processing time (minutes). * $p < 0.05$. There were no significant differences after correction for multiple testing (FDR = 0.2).

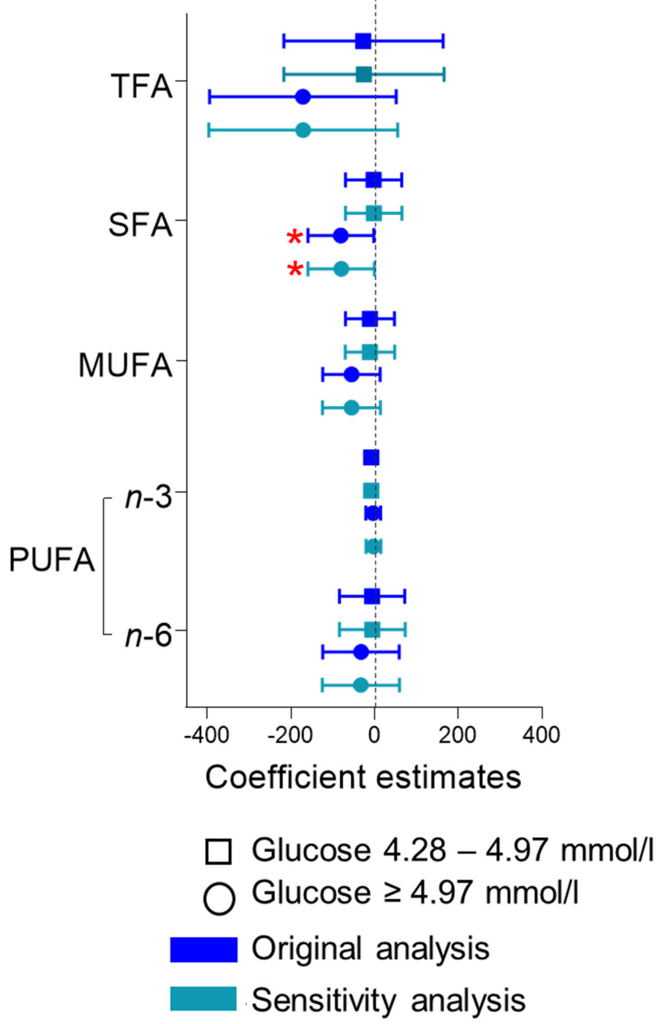

Supplement: Supplementary file 1 [file ijms-22-10422-s001.zip › ijms-1364751-supplementary.pdf]
